# The alfa and beta of tumours: a review of parameters of the linear-quadratic model, derived from clinical radiotherapy studies

**DOI:** 10.1186/s13014-018-1040-z

**Published:** 2018-05-16

**Authors:** C. M. van Leeuwen, A. L. Oei, J. Crezee, A. Bel, N. A. P. Franken, L. J. A. Stalpers, H. P. Kok

**Affiliations:** 1 Department of Radiation Oncology, Academic Medical Center, University of Amsterdam, Meibergdreef 9, 1105 Amsterdam, AZ The Netherlands; 2 Laboratory for Experimental Oncology and Radiobiology (LEXOR)/Center for Experimental Molecular Medicine, Academic Medical Center, University of Amsterdam, Amsterdam, The Netherlands

**Keywords:** α/β ratio, Fractionation sensitivity, Radiosensitivity, Study heterogeneity

## Abstract

**Background:**

Prediction of radiobiological response is a major challenge in radiotherapy. Of several radiobiological models, the linear-quadratic (LQ) model has been best validated by experimental and clinical data. Clinically, the LQ model is mainly used to estimate equivalent radiotherapy schedules (e.g. calculate the equivalent dose in 2 Gy fractions, EQD_2_), but increasingly also to predict tumour control probability (TCP) and normal tissue complication probability (NTCP) using logistic models. The selection of accurate LQ parameters α, β and α/β is pivotal for a reliable estimate of radiation response. The aim of this review is to provide an overview of published values for the LQ parameters of human tumours as a guideline for radiation oncologists and radiation researchers to select appropriate radiobiological parameter values for LQ modelling in clinical radiotherapy.

**Methods and materials:**

We performed a systematic literature search and found sixty-four clinical studies reporting α, β and α/β for tumours. Tumour site, histology, stage, number of patients, type of LQ model, radiation type, TCP model, clinical endpoint and radiobiological parameter estimates were extracted. Next, we stratified by tumour site and by tumour histology. Study heterogeneity was expressed by the I^2^ statistic, i.e. the percentage of variance in reported values not explained by chance.

**Results:**

A large heterogeneity in LQ parameters was found within and between studies (I^2^ > 75%). For the same tumour site, differences in histology partially explain differences in the LQ parameters: epithelial tumours have higher α/β values than adenocarcinomas. For tumour sites with different histologies, such as in oesophageal cancer, the α/β estimates correlate well with histology. However, many other factors contribute to the study heterogeneity of LQ parameters, e.g. tumour stage, type of LQ model, TCP model and clinical endpoint (i.e. survival, tumour control and biochemical control).

**Conclusions:**

The value of LQ parameters for tumours as published in clinical radiotherapy studies depends on many clinical and methodological factors. Therefore, for clinical use of the LQ model, LQ parameters for tumour should be selected carefully, based on tumour site, histology and the applied LQ model. To account for uncertainties in LQ parameter estimates, exploring a range of values is recommended.

**Electronic supplementary material:**

The online version of this article (10.1186/s13014-018-1040-z) contains supplementary material, which is available to authorized users.

## Background

Prediction of biological response after irradiation has been a challenge since the discovery of X-rays and radium. In the early days of radiotherapy it became clear that the biological effect of irradiation was not only determined by the total dose, but also by the characteristics of the treatment schedule such as fraction dose, dose rate and overall treatment time [1]. Many models have been proposed to predict radiobiological response. The linear-quadratic (LQ) model has been best validated by experimental and clinical data, and its conceptual simplicity added to its present popularity in radiotherapy practice, for instance to address clinical problems such as compensation for missed treatment days, comparison of different treatment schemes, and the design of novel treatment schedules in clinical trials [2–4].

The basic LQ model describes the surviving fraction *SF* of clonogenic or stem cells as a function of radiation dose *D,*

1 $$ SF(D)={e}^{-\alpha \bullet D-\beta \bullet {D}^2} $$

The main parameters of this model, *α* and *β*, represent the intrinsic radiosensitivity of the irradiated cells: cells with a higher *α* and *β* are more sensitive to radiation. The ratio of the two parameters, α/β, is a measure of the fractionation sensitivity of the cells: cells with a higher α/β, are less sensitive to the sparing effect of fractionation. Several extensions to the basic LQ model have been developed, particularly to account for incomplete repair [5] and repopulation [6]. The LQ model has shown its clinical usefulness in predicting the sparing effect of fractionated radiotherapy, and in comparing the equivalent total dose of different fractionation schedules. The estimation of radiotherapeutic outcome, and therapeutic window strongly depends on a reliable estimation of LQ parameters α, β and α/β.

The radiation sensitivity parameters α and β can be measured in vitro in tumour cell lines, but artificial cell line cultures may not be representative for clinical radiobiological calculations. Under some model assumptions, α and β can be derived from clinical radiotherapy data, i.e. from the tumour control probability (TCP), by fitting the TCP for different radiotherapy schedules to a logistic- or Poisson-like TCP model. Alternatively, the α/β ratio can be inferred from two or more iso-effective fractionation schedules, as originally described by Thames et al. [7].

Many studies have estimated these radiobiological LQ parameters from clinical data for different tumour sites [5, 8–70]. There are a few publications wherein aggregate data for α/β have been presented in tables for different tumour sites [4, 51], but these reviews did only report values for a limited number of sites, did only include one single study per tumour site and did not report separate α and β values. Reviews and meta-analyses of multiple fractionation trials have also been published, but only for prostate [59] and breast cancer [33]. Therefore, we wish to give a more comprehensive overview of published LQ parameters for all tumour sites and all histologies.

Several issues arise when collecting radiobiological parameters of human tumours from the literature. Some studies have published LQ parameters as a main objective, but reported radiobiological parameters are often hidden in a paper wherein the assessment of a radiobiological parameter had not been the primary goal of a clinical study. Next, different literature values may be reported for the same tumour site, even for the same study population (e.g. [19, 22, 38, 70]), making it difficult to know which value is appropriate for the situation of interest.

Another important challenge is *study heterogeneity*, which is the variation in LQ parameter values that cannot be explained by chance, i.e. a variation that is larger than expected by the reported variance and/or confidence intervals. Study heterogeneity should not be confused with intratumour and intertumour heterogeneity, which are well known and can be dealt with by explicit modelling of such heterogeneity (e.g. [71–73]). The presence of study heterogeneity indicates that studies are not estimating a single outcome (e.g. α/β value), but that each study estimates a value which is only valid for the specific method and patient cohort of that specific study. Study heterogeneity is a well-known pitfall of literature reviews; the Cochrane Institute recommends to quantify study heterogeneity and explore its origin, rather than to perform a meta-analysis [74].

Thus, the aim of this review is to give an overview of published values for the LQ parameters α, β and α/β of human tumours, to quantify study heterogeneity of these values, and to identify possible causes of study heterogeneity. Thereby, we wish to provide a guideline for radiation oncologists and radiation researchers to select appropriate radiobiological parameter values for LQ modelling in clinical radiotherapy.

## Methods

### Search & inclusion criteria

Relevant studies were identified from the Medline database using PubMed with combinations of the search terms “dose-response relationship, radiation”, “dose fractionation”, “linear”, “quadratic”, “alpha”, “beta” and “humans” (see Additional file 1: Appendix S1 for the full search strategy). The search includes studies indexed until January 24, 2017 and was limited to articles in English.

Studies on patients with any tumour were eligible, regardless of tumour site or histology. Intervention needed to have included radiotherapy with photons; no limitations were imposed on the radiation type (external beam irradiation, brachytherapy), radiation technique (3D conformal, IMRT, etc.) or adjuvant treatments. Studies needed to have estimated values for α, β or α/β. No limitation was imposed on the clinical endpoint (e.g. local control, survival, biochemical control) on which these estimates were based. Studies wherein multiple analyses were performed, and different studies which analysed the same clinical data set (but using different methods or different subsets of the clinical data set), were included as separate analyses. We excluded studies wherein either α, β or a/β were fixed in the fitting procedure.

Clinical variables that were extracted were tumour site, histology, stage, radiation type (external beam radiotherapy and/or brachytherapy), and number of patients. Methodological variables that were extracted were the type of LQ model (e.g. basic or accounting for repopulation), TCP model (e.g. Poisson or logistic) and clinical endpoint used to derive the radiobiological parameters (see Additional file 1: Table S5). Finally, the LQ parameter estimates were extracted, including their confidence intervals and/or variances, when reported. Studies not reporting confidence intervals or variances were still included, but marked as such in the relevant tables. This was deemed justified since we aim for a review of LQ parameter estimates as complete as possible and for some tumor categories these studies not reporting confidence intervals or variances represented the only available data.

### Statistical analysis

Separate overviews were made for α, β and α/β. Two different stratifications were made, by tumour site and by histology. For each stratum containing at least two radiobiological parameter estimates, study heterogeneity was quantified for each of the three parameters (α, β and α/β) using the *I*^*2*^ statistic, as recommended by the Cochrane Institute [74]. The I^2^ statistic represents the percentage of variance in reported LQ parameters that is not explained by chance and which is therefore due to clinical or methodological differences between studies [75].

Categorization is not strict, but I^2^ values of 25, 50 and 75% are usually considered as low, moderate and high heterogeneity, respectively [75]. To calculate I^2^, the variance of the reported outcome (α, β or a/β) is needed. If a study reported the variance, this was directly used to calculate I^2^. Otherwise, the variance was estimated from the 95% confidence interval. Analyses in which neither the variance, nor the confidence interval was reported were not included in the calculation of I^2^.

It is debated whether the LQ model is still valid at large fraction sizes [76–78]. If not, the inclusion of patients treated with brachytherapy could lead to different radiobiological parameter estimates, as brachytherapy fraction sizes are typically large. To investigate the possibility that heterogeneity in radiobiological parameter estimates was (partly) caused by the inclusion of data from patients treated with brachytherapy, I^2^ was also calculated on the subset of studies that only included data from patients treated with external beam radiotherapy.

## Results

**Fig. 1:**
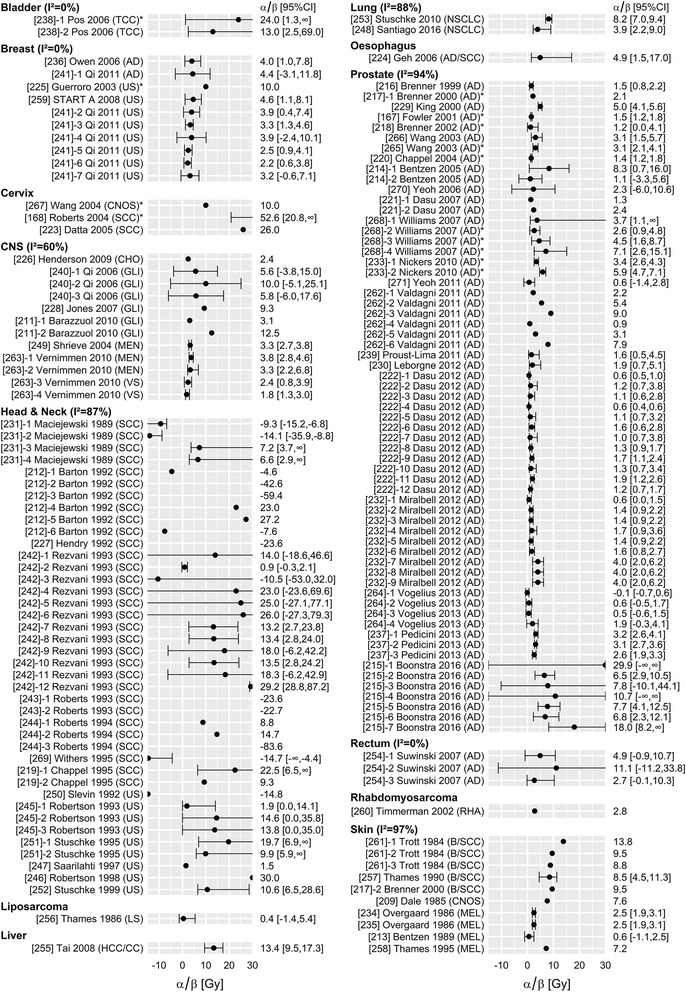
Overview of 149 reported estimates of a/β, stratified by tumour site. Within tumour sites, studies are sorted by histology, and then by date of publication. TCC: transitional cell carcinoma; AD: adenocarcinoma; US: unspecified; CNOS: carcinoma, not otherwise specified; SCC: squamous cell carcinoma; CHO: chordoma; GLI: glioma; MEN: meningioma; VS: vestibular schwannoma; LS: liposarcoma; HCC/CC: Hepatocellular carcinoma & Cholangiocarcinoma; NSCLC: Non small cell lung carcinoma; RHA: Rhabdomyosarcoma; B/SCC: Basal-cell carcinoma & Squamous cell carcinoma; MEL: melanoma. *Included data of patients treated with brachytherapy as part of the treatment. N.B. [56] Withers 1995 reported a 95% confidence interval consisting of two segments, (− 8,-4.4) and (13.7,8)

**Fig. 2:**
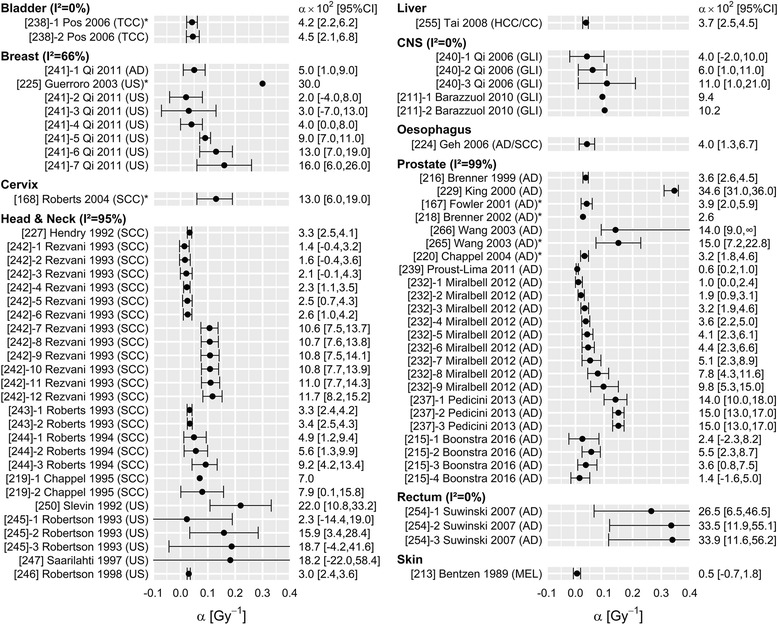
Overview of 72 reported estimates of a, stratified by tumour site. Within tumour sites, studies are sorted by histology, and then by date of publication. TCC: transitional cell carcinoma; AD: adenocarcinoma; US: unspecified; SCC: squamous cell carcinoma; GLI: glioma; HCC/CC: Hepatocellular carcinoma & Cholangiocarcinoma; MEL: melanoma. *Included data of patients treated with brachytherapy as part of the treatment

**Fig. 3:**
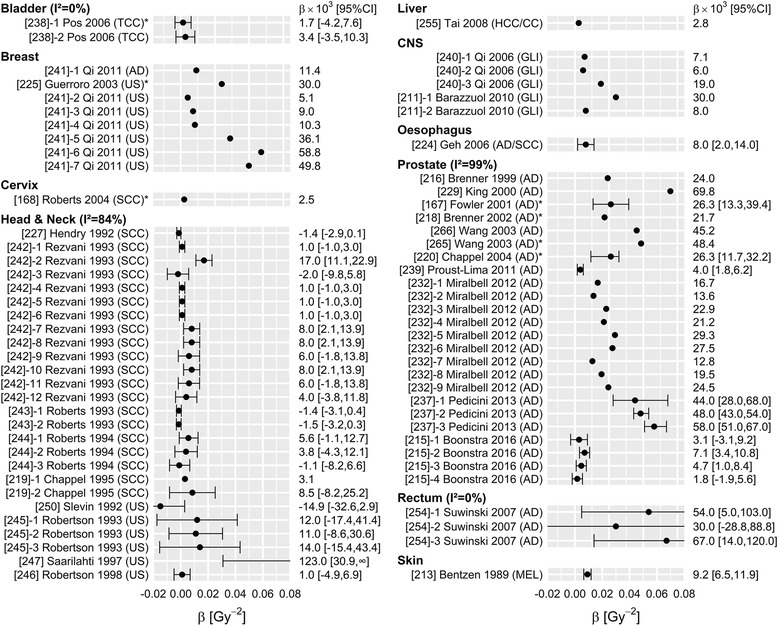
Overview of 72 reported estimates of β, stratified by tumour site. Within tumour sites, studies are sorted by histology, and then by date of publication. TCC: transitional cell carcinoma; AD: adenocarcinoma; US: unspecified; SCC: squamous cell carcinoma; GLI: glioma; HCC/CC: Hepatocellular carcinoma & Cholangiocarcinoma; MEL: melanoma. *Included data of patients treated with brachytherapy as part of the treatment

The initial literature search yielded 1177 papers of which eventually 64 satisfied our inclusion criteria [5, 8–70] (for the PRISMA flow diagram, see Additional file 1: Figure S2). These 64 papers reported 149 different analyses of α/β based on 81 distinct sets of clinical data (Fig. 1). For α and β, 72 different analyses were found based on 39 distinct sets of clinical data (Figs. 2 and 3). Similar figures for the stratification by tumour histology may be found in the Additional file 1: Figure S3.1-S3.3.

Either variance or confidence interval was reported in 67, 42 and 110 analyses of α, β and α/β respectively, and these analyses were used to quantify study heterogeneity (I^2^). Study heterogeneity was substantial for all three parameters (Figs. 1, 2, 3), particularly in those strata that contain many analyses (head and neck and prostate tumour sites; adenocarcinoma and squamous cell carcinoma histologies). For example, I^2^ estimates of α/β for tumours of the head & neck, prostate and skin were 87, 94 and 97%, respectively. The α/β estimates for breast, bladder and rectum cancer were an exception: heterogeneity within those strata was extremely low. This is most likely due to the fact that (almost) all studies included in those strata were performed by the same author, excluding heterogeneity due to methodological differences. I^2^ values were not substantially different when studies including data from patients treated with brachytherapy were excluded (Additional file 1: Table S6).

Despite study heterogeneity, a number of patterns could be identified. Estimates of α/β for prostate tumours, breast tumours, rhabdomyosarcoma and liposarcoma generally indicated a high fractionation sensitivity (mostly, α/β≈4 Gy), although only a single estimate was available for the latter two. Estimates of α/β for head & neck, cervix, bladder and liver tumours generally indicated low fractionation sensitivity (mostly, α/β = 10 Gy or α/β = − 10 Gy), with only limited data being available for the latter three. Estimates of α/β for rectum, oesophagus, central nervous system (CNS), skin and lung tumours were very mixed. This is probably related to the very different histologies that occur at these sites. For the central nervous system, Henderson [16], Shrieve [43] and Vernimmen [58] studied benign histologies (chordoma, meningioma and vestibular schwannoma), and all reported low α/β estimates (≈4 Gy). Qi [32], Jones [18] and Barazzuol [8] studied various types of glioma, and generally found intermediate α/β values (typically 5–10 Gy). Similarly in skin, estimates for melanoma (Overgaard [25, 26], Bentzen [30], Thames [53] were low (< 3 Gy, with one exception), while estimates for mixed basal-cell and squamous cell skin tumours were high (≈10 Gy). For lung, the α/β estimates were based on cohorts with mixed histologies, and different histological composition of those cohorts may explain the differences between those two studies.

Estimates for *α* were mostly in the range of 0.02–0.2 Gy^− 1^, and no striking differences were found between tumour sites. The value of *α* appeared somewhat higher for rectal cancer, but this may be the result of the specific methods applied in that study; Suwinski [48] investigated a group of patients who also had surgery, and included a term accounting for the tumour control probability through surgery alone. An educated guess was made for this tumour control probability, and any change in that estimate may have substantially affected the value of *α* (and *β*) that was found. Estimates of *β* vary from 0.001–0.06 Gy^− 2^ and appear to be somewhat higher for breast and prostate tumours, which corresponds with the lower α/β values found for these sites.

The main clinical characteristics (number of patients, tumour stage, radiation type (external beam radiotherapy and/or brachytherapy), site and histology) and methodological characteristics, (type of LQ model, type of TCP model and clinical endpoint) of the included analyses can be found in the Additional file 1: Table S4. In most cases either the basic LQ model, or an LQ model with a correction for repopulation was used. Less frequently, also a term was included to account for repair of sublethal damage, which is relevant only for protracted irradiation. The type of LQ model can substantially affect the radiobiological parameters. This was clearly demonstrated by Suwinski et al. [48], who fitted the same rectal cancer data both with and without a time factor to account for repopulation. The estimated α/β value without time factor was 5.1 Gy (i.e. relatively sensitive to fractionation), but introduction of a time factor increased the same LQ parameter to 11.1 Gy (i.e. relatively insensitive to fractionation).

Poissonian or logistic TCP models were the most commonly used models to relate the cell survival fraction predicted by the LQ model to a clinical outcome parameter (e.g. local tumour control). Less common was the use of a Cox proportional hazards model. In analyses in which the individual *α* and *β* were not estimated, α/β was often estimated based on two (or more) iso-effective treatment schedules, in which case an explicit TCP model is not needed. A short description of the most-used LQ and TCP models is given in the Additional file 1: Table S5.

## Discussion

The LQ model is increasingly being used to predict control probability (TCP) and normal tissue complication probability (NTCP) using logistic models, for instance for radiobiological treatment planning [79–81]. In this study we summarized published values for the LQ parameters *α, β* and α/β of human tumours, for as many tumour sites and tumour histologies as possible. This overview shows a large study heterogeneity in reported values of LQ parameters, which indicates substantial clinical and methodological differences between studies. Despite study heterogeneity, some relevant patterns could be identified.

Commonly, α/β values are categorized by tumour site [4, 82], implicitly assuming that tumour site is the most important factor determining radiobiological behaviour. The rationale for categorization by tumour site is that clinical radioresponsiveness would predominantly be determined by the tumour environment (e.g. hypoxia). However, Fertil and Malaise [83] already showed in 1985 that radiosensitivity is (at least partly) intrinsic to the histology of the tumour. Our data support that both tumour site and histology independently determine radioresponsiveness. The first idea, that tumour site is important, is supported by the fact that prostate tumours seem to have even lower α/β (±1–2 Gy) than breast tumours (±2–4.5 Gy), even though both are adenocarcinoma. The second idea, that tumour histology is an independent important factor, is supported by the observation that similar histologies show consistently similar α/β values, regardless of tumour site. Adenocarcinomas, both in prostate and breast cancer, overall display a high fractionation sensitivity (low α/β, see Additional file 1: Figure S3.1). On the other hand, epithelial histologies such as squamous cell carcinoma, transitional cell carcinoma, basal cell carcinoma and non small cell lung carcinoma all exhibit low fractionation sensitivity (high α/β). Finally, some tumour sites (e.g. skin and central nervous system tumours) exhibit very mixed fractionation sensitivities that correlate well with the different histologies occurring at those sites. In summary, both site and histology are important factors for α/β. Therefore, it has been suggested that for tumour sites at which multiple histologies occur (e.g. squamous-cell carcinoma and adenocarcinoma in oesophageal cancer), LQ parameters should be reported separately for each histology [14], which enables estimation of separate α/β values for each histology. This finding may be relevant for LQ calculations in radiotherapy practice, for instance in a patient with cancer of unknown origin, or for a patient with a tumour in a site with more histologies (i.e. lung, oesophagus, cervix uteri), for whom we recommend to choose an α/β based on the tumour histology.

Apart from tumour site and histology, the type of LQ model used in an analysis may affect the values estimated for α, β and a/β and thus partially explains study heterogeneity. For example, in the study by Suwinski et al. [48] a higher α/β was reported when a time factor was included (α/β = 11.1 Gy) than without time factor (α/β = 5.1 Gy). This can be explained by the fact that high dose-per-fraction treatment schedules are often shorter than low dose-per-fraction schedules. Therefore, when using a time factor to account for repopulation, part of the efficacy of a high-dose-per fraction schedule is attributed to a shortened overall treatment time, and not to the higher fraction dose. Then, the inclusion of a time factor will result in a higher estimate for α/β. Another example is that the estimates for α and β are higher when intratumour heterogeneity is accounted for in the LQ model [20]. This is because these values represent the mean radiosensitivity, while the tumour control is mostly determined by the most radioresistant (i.e. low α and β) tumour cells within the tumour.

Due to statistical variation, some studies find small, negative β estimates. As a result, large, negative values are calculated for α/β (e.g. [65]). This is merely a statistical effect: regardless of the sign, a small absolute value β (and correspondingly large absolute value of α/β) indicates that the tumour has a very low sensitivity to the effects of fractionation. Although from radiobiological point of view negative values of the α/β ratio are not realistic, it is not advised to constrain negative values in radiobiological analyses. When parameters are constrained, aggregate estimates do not converge to the true value. Furthermore, constraining parameters in e.g. maximum likelihood regression results in inaccurate estimates of the confidence intervals. Withers et al. [65] suggested to use β/α instead of α/β [65], since β/α has better balanced statistical properties. While statistical variation could still result in negative β/α estimates, these now have a more intuitive interpretation: all tumours with β/α close to zero have a low fractionation sensitivity, while tumours with a large β/α are sensitive to fractionation. Nevertheless, the α/β-ratio remained the standard LQ parameter for fractionation sensitivity.

Prior to this study, Qi et al. [33] did a meta-analysis on breast cancer, and Vogelius et al. [59] on prostate cancer. Vogelius et al. [59] determined α/β based on five prostate cancer studies (including 1965 patients), and showed that within their data no heterogeneity was present (I^2^ = 0%). Qi et al. [33] determined both α/β and *α* based on seven aggregated studies (including 8269 patients). They did not calculate study heterogeneity, but all data required for heterogeneity calculation were reported. For α/β, no heterogeneity was present (I^2^ = 0%), while for α heterogeneity was substantial (I^2^ = 58%). For the majority of tumour sites in our study, study heterogeneity in α/β was substantially higher than what was found in these two studies. This difference is most likely due to the specific design of these studies, which excluded several potential sources of heterogeneity. For example, Vogelius et al. only included studies in which external radiotherapy was the primary treatment for prostate cancer (i.e. no brachytherapy or prior prostatectomy), thereby excluding these potential sources of clinical heterogeneity. Furthermore, rather than aggregating available radiobiological parameters, these two meta-analyses used local control and biochemical control of PSA from fractionation trials to derive LQ parameters for each individual trial. As a result, the LQ parameter estimates were derived using exactly the same statistical analysis, excluding potential sources of methodological heterogeneity. This approach is unfortunately only feasible for those tumour sites where many fractionation trials have been performed. Moreover, due to the strict inclusion criteria of Vogelius et al., their results are only applicable to a very specific patient group, whereas our study aimed to present a complete overview of the available data.

Qi et al. [33] and Vogelius et al. [59] previously reported meta-analyses of LQ parameters for uniformly treated patients with breast cancer and prostate cancer respectively, both yielding relatively low α/β values for tumour, and low study heterogeneities. Their results are only applicable to two specific patient groups. We chose to aggregate LQ parameter estimates for as many sites and histologies as possible, at the cost of a higher study heterogeneity. To select radiobiological parameters from this overview, one should try to select parameters from a study that matches the situation of interest (both clinically and methodologically) as close as possible (see Additional file 1: Table S4). We recommend this elaborate approach for the selection of LQ parameters in the design of a radiobiological treatment planning system.

However, for LQ calculations in daily radiation practice, we recommend to use a range of plausible α/β values rather than a single value. A plausible range can be found in Figs. 1, 2, 3. For example, when selecting an α/β for breast tumours, the radiobiological calculation could be performed with α/β = 2 Gy, 3.5 Gy and 5 Gy respectively. If a consistent conclusion can be drawn for the whole range of plausible values (e.g. one schedule is more effective than another, for all three α/β values), this conclusion may be considered robust to the uncertainty in the selection of appropriate parameters. This approach is valid irrespective of the estimated heterogeneity, although the range of plausible values is likely to be larger for tumour sites with larger heterogeneity.

## Conclusions

This review provides an overview of published values for the LQ parameters of human tumours as a guideline for radiation oncologists and radiation researchers to select appropriate radiobiological parameter values for LQ modelling in clinical radiotherapy. The estimation of LQ parameters for tumours in published clinical radiotherapy studies was subject to many clinical and methodological factors, which explain the wide range of reported values. Therefore, for LQ calculations in radiotherapy practice, the α/β ratio of tumour should be selected carefully, based on tumour site, tumour histology and the applied LQ model. To account for uncertainties in LQ parameter values, it is recommended to explore a range of plausible α/β values.

## Additional file

Additional file 1: **Appendix S1.** Search strategy. **Figure S2.** PRISMA flow chart. **Figure S3.** Forest plots of α, β and α/β, stratified by tumor histology. **Tables S4.** Characteristics of included studies. **Table S5.** Common LQ models and TCP models. **Table S6.** Heterogeneity with/without studies including brachytherapy. (PDF 1223 kb)

## Abbreviations

LQ: Linear-quasdratic
NTCP: Normal tissue complication probability
SF: Surviving fraction
TCP: Tumour control probability
