# Supplementary material for: The alfa and beta of tumours: a review of parameters of the linear-quadratic model, derived from clinical radiotherapy studies
Source: Radiat Oncol. 2018 May 16;13:96. doi: 10.1186/s13014-018-1040-z (PMC5956964; doi:10.1186/s13014-018-1040-z)
Supplement: Supplementary file 1 — Appendix S1. Search strategy. Figure S2. PRISMA flow chart. Figure S3. Forest plots of α, β and α/β, stratified by tumor histology. Tables S4. Characteristics of included studies. Table S5. Common LQ models and TCP models. Table S6. Heterogeneity with/without studies including brachytherapy. (PDF 1223 kb) [file 13014_2018_1040_MOESM1_ESM.pdf]

Additional file 1: Appendix S1 – Search strategy

Search strategy: Medline (PubMed)

1. dose-response relationship, radiation[MeSH Terms]
2. dose fractionation[MeSH Terms]
3. 1 OR 2
4. Linear[All Fields]
5. quadratic[All Fields])
6. 4 AND 5
7. alpha[All Fields]
8. beta[All Fields]
9. 7 AND 8
10. 6 OR 9
11. 3 AND 10
12. Linear[All Fields]
13. quadratic[All Fields]
14. 12 AND 13
15. LQ[All Fields]
16. 14 OR 15
17. alpha[All Fields]
18. beta[All Fields]
19. 16 AND 17 AND 18
20. 11 OR 19
21. humans[MeSH Terms]
22. English[LA]
23. 20 AND 21 AND 22

Additional file 1: Figure S2 – PRISMA flow chart

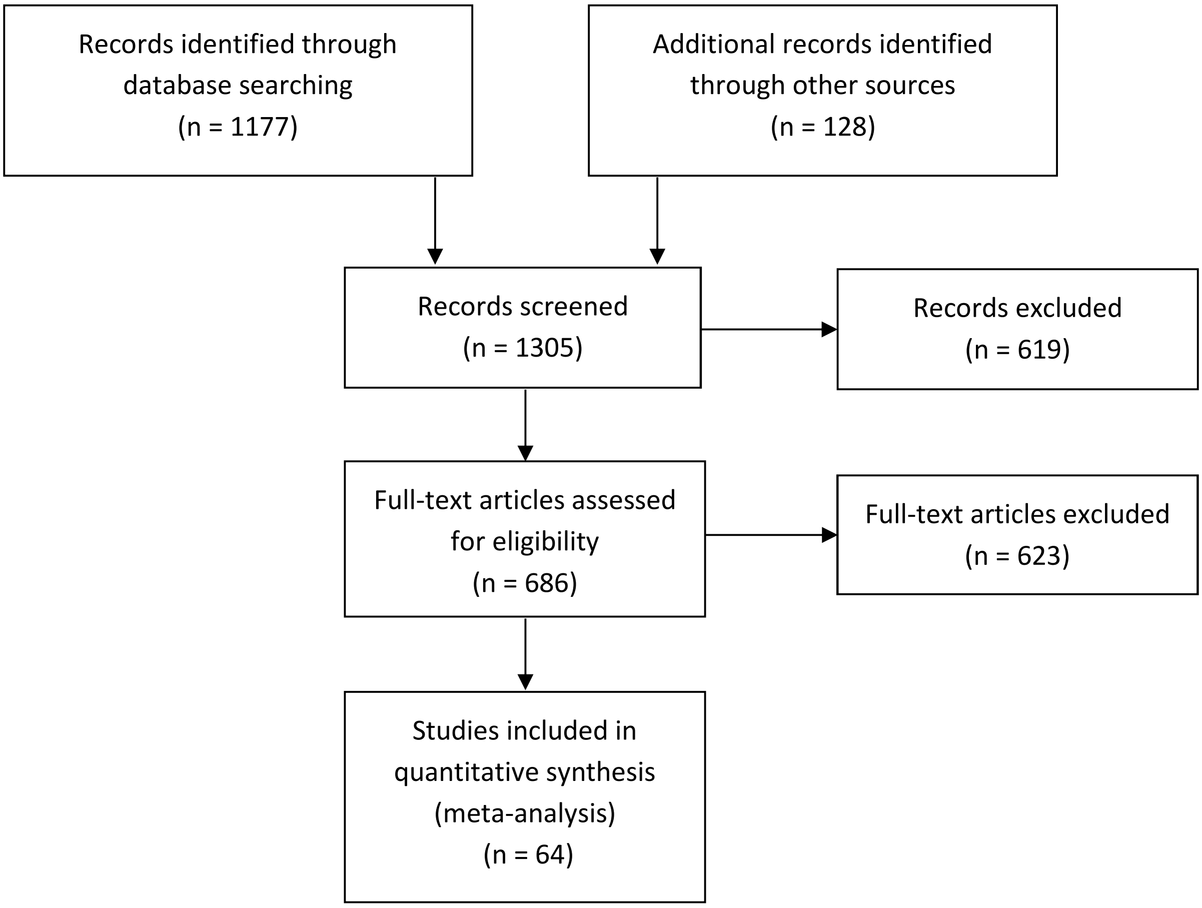

Figure S2. PRISMA flow chart. Because determination of radiobiological parameters is seldom the primary goal of a study, an unconventionally large number of papers passed initial title/abstract screening and could only be excluded after full-text analysis. Most common reasons for exclusion where that studies used radiobiological parameters in an analysis, but did not derive them, or that studies were based on animal or cell line data.

Additional file 1: Figure S3 – Forest plots of α, β and α/β, stratified by tumor histology

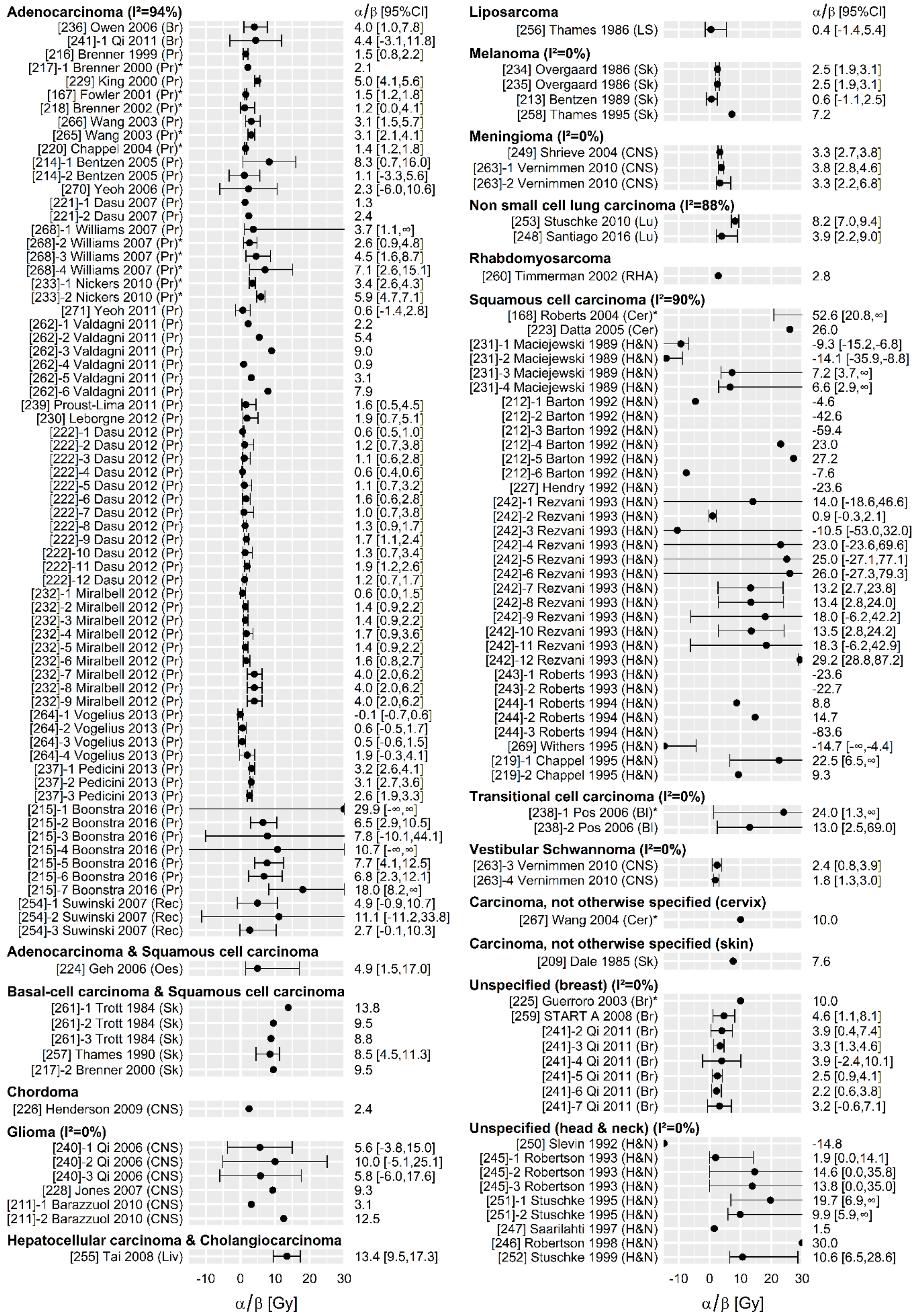

Figure S3.1. Overview of 149 reported estimates of α/β, stratified by tumor histology. Within tumor histologies, studies are

sorted by tumor site, and then by date of publication. Br: breast; Pr: prostate; Rec: rectum; Oes: oesophagus; Sk: skin; CNS: central nervous system; Liv: liver; LS: liposarcoma; Lu: Lung; RHA: rhabdomyosarcoma; Cer: cervix; H&N: head & neck; Bl: bladder. *Included data of patients treated with brachytherapy as part of the treatment. N.B. [68] Withers 1995 reported a 95% confidence interval consisting of two segments, (-∞,-4.4) and (13.7,∞).

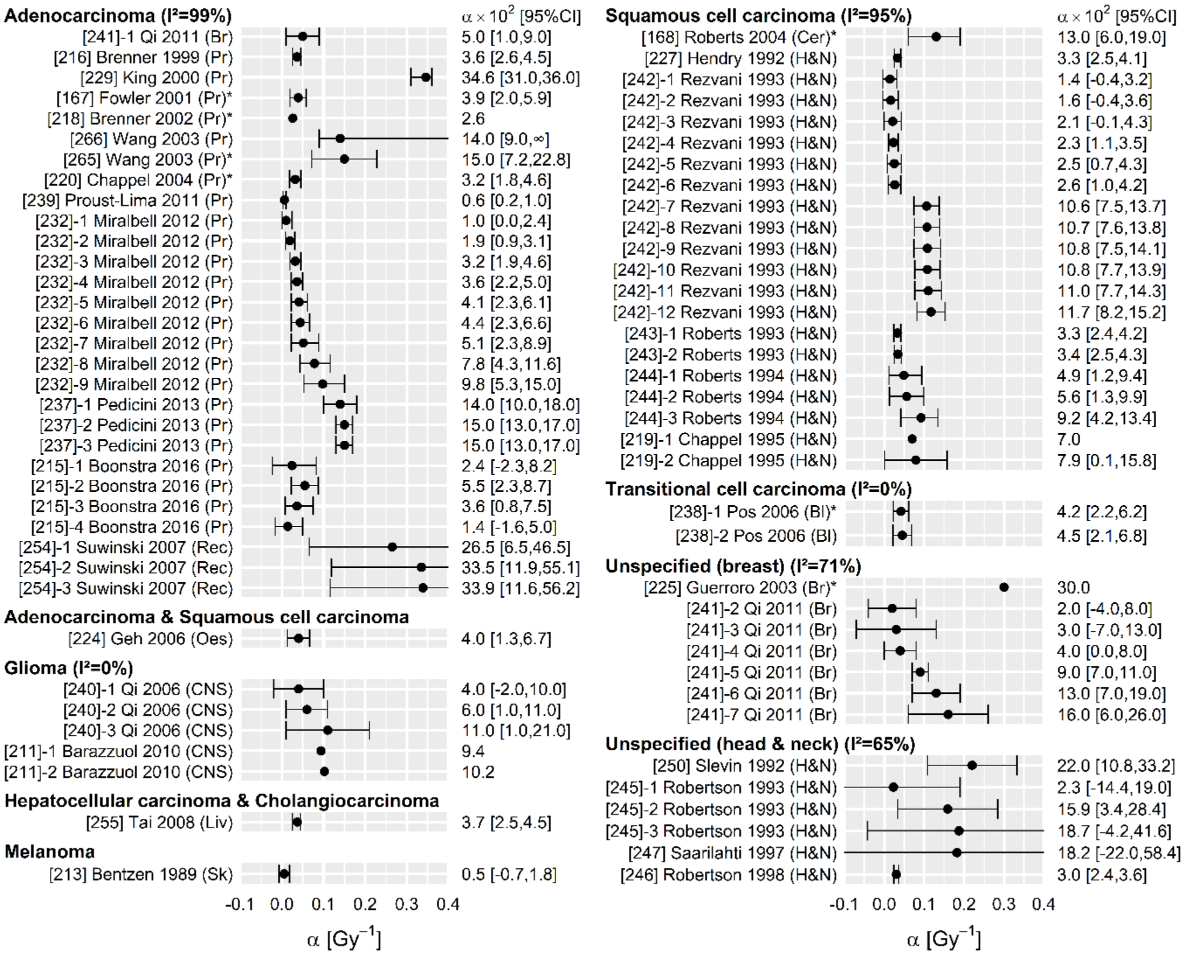

Figure S3.2. Overview of 72 reported estimates of α, stratified by tumor histology. Within tumor histologies, studies are sorted by tumor site, and then by date of publication. Br: breast; Pr: prostate; Rec: rectum; Oes: oesophagus; CNS: central nervous system; Liv: liver; Sk: skin; Cer: cervix; H&N: head & neck; Bl: bladder. *Included data of patients treated with brachytherapy as part of the treatment.

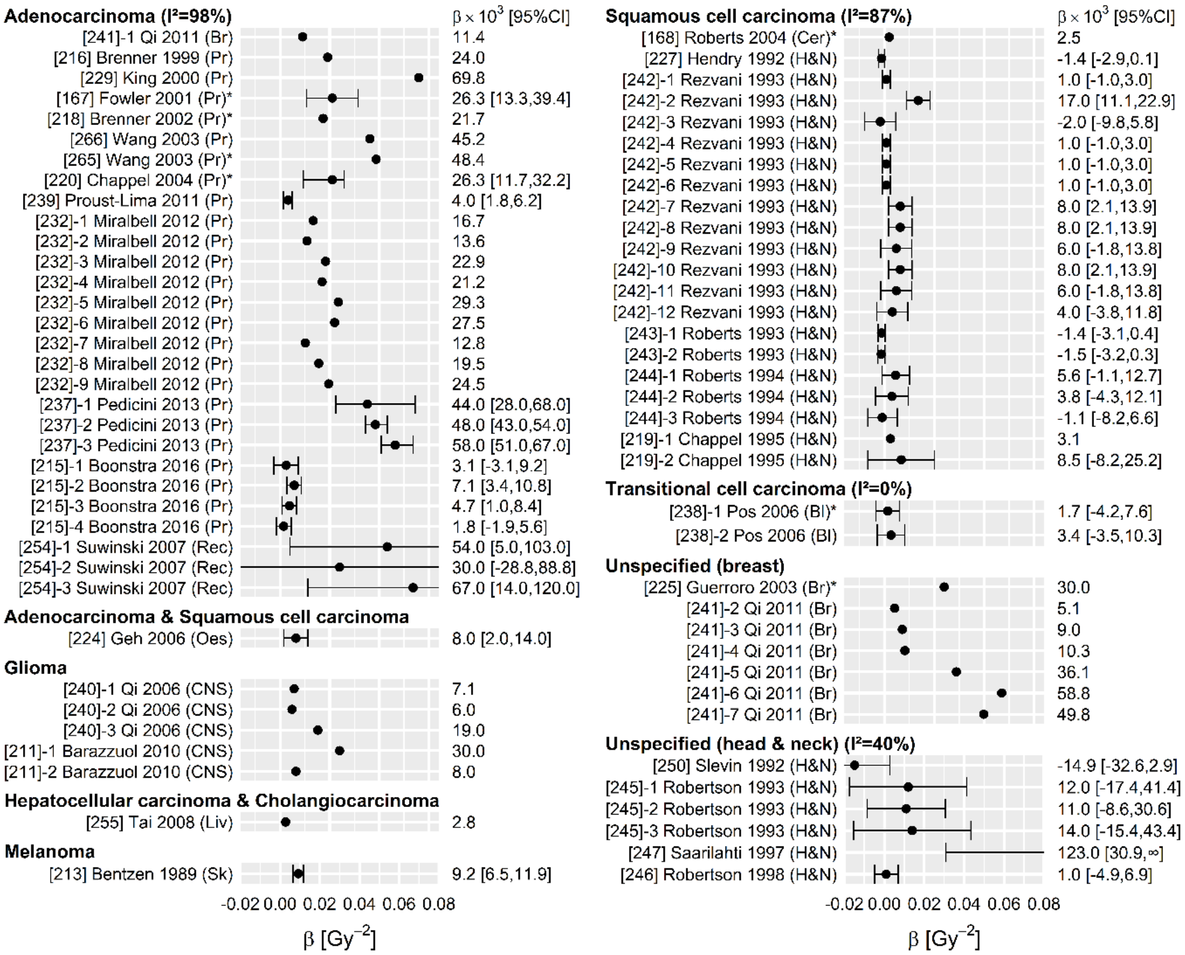

Figure S3.3. Overview of 72 reported estimates of β, stratified by tumor histology. Within tumor histologies, studies are sorted by tumor site, and then by date of publication. Br: breast; Pr: prostate; Rec: rectum; Oes: oesophagus; CNS: central nervous system; Liv: liver; Sk: skin; Cer: cervix; H&N: head & neck; Bl: bladder. *Included data of patients treated with brachytherapy as part of the treatment.

Additional file 1: Table S4 – Characteristics of included studies

Table S4

Summary of characteristics of the included studies, along with the reported values for α, β and α/β. The table is sorted by site; Ties are broken by histology, then year of publication. Reference and sequence numbers correspond to those in Figures 1-3 and Figures S3.1-S3.3.

**Study**	**#Patients**	**Stages**	**Site**	**Histology**	**LQ model**	**TCP model**	**Endpoint**	**α [Gy^{-1}]**	**β [Gy^{-2}]**	**α/β [Gy]**
[36]-1 Pos 2006*	1302	T1-T4	Bladder	Transitional cell carcinoma	LQ+RP	Logistic	Local control	0.042	0.0017	24
[36]-2 Pos 2006	833	T1-T4	Bladder	Transitional cell carcinoma	LQ (basic)	Logistic	Local control	0.045	0.0034	13
[34] Owen 2006	1410	TisN0-T2N1	Breast	Adenocarcinoma	LQ (basic)	Cox	Local control	X	X	4
[39]-1 Qi 2011	1410	TisN0-T2N1	Breast	Adenocarcinoma	LQ+RPP	Poisson	Local control	0.05	0.0114	4.39
[23] Guerroro 2003*	1005	X	Breast	Unspecified	LQ+RP+RPP	Poisson	Local control	0.3	0.03	10
[58] START A 2008	3646	T1N0M0-T3N1M0	Breast	Unspecified	LQ (basic)	Cox	Locoregional control	X	X	4.6
[39]-2 Qi 2011	2236	T1N0M0-T3N1M0	Breast	Unspecified	LQ+RPP	Poisson	Locoregional control	0.02	0.0051	3.91
[39]-3 Qi 2011	837	X	Breast	Unspecified	LQ+RPP	Poisson	Local control	0.03	0.009	3.34
[39]-4 Qi 2011	453	X	Breast	Unspecified	LQ+RPP	Poisson	Local control	0.04	0.0103	3.89
[39]-5 Qi 2011	2215	T1N0M0-T3N1M0	Breast	Unspecified	LQ+RPP	Poisson	Locoregional control	0.09	0.0361	2.49
[39]-6 Qi 2011	292	T1N0-T2N1	Breast	Unspecified	LQ+RPP	Poisson	Local control	0.13	0.0588	2.21
[39]-7 Qi 2011	1234	I-III	Breast	Unspecified	LQ+RPP	Poisson	Local control	0.16	0.0498	3.21
[66] Wang 2004*	541	I-IV	Cervix	Carcinoma (NOS)	LQ (basic)	None	Locoregional control	X	X	10
[43] Roberts 2004*	517	I-II	Cervix	Squamous cell carcinoma	LQ+RP+RPP	Poisson	Local control	0.13	0.0025	52.63
[20] Datta 2005	77	I-IV	Cervix	Squamous cell carcinoma	LQ+RPP	None	Tumor regression	X	X	26
[24] Henderson 2009	86	X	CNS	Chordoma	LQ (basic)	None	Local control	X	X	2.45
[38]-1 Qi 2006	243	IV	CNS	Glioma	LQ+RP+RPP	Poisson	Survival	0.04	0.0071	5.6
[38]-2 Qi 2006	864	X	CNS	Glioma	LQ+RP+RPP	Poisson	Survival	0.06	0.006	10
[38]-3 Qi 2006	243	III	CNS	Glioma	LQ+RP+RPP	Poisson	Survival	0.11	0.019	5.8
[26] Jones 2007	443	III-IV	CNS	Glioma	LQ+RPP	None	Regrowth delay	X	X	9.32
[8]-1 Barazzuol 2010	187	X	CNS	Glioma	LQ+RPP	None	Survival	0.094	0.03	3.1

[8]-2 Barazzuol 2010	187	X	CNS	Glioma	LQ+RPP	None	Survival	0.102	0.008	12.5
[48] Shrieve 2004	X	X	CNS	Meningioma	LQ (basic)	None	Local control	X	X	3.28
[62]-1 Vernimmen 2010	1175	X	CNS	Meningioma	LQ (basic)	None	Local control	X	X	3.76
[62]-2 Vernimmen 2010	1175	X	CNS	Meningioma	LQ (basic)	None	Local control	X	X	3.3
[62]-3 Vernimmen 2010	1897	X	CNS	Vestibular Schwannoma	LQ (basic)	None	Local control	X	X	2.4
[62]-4 Vernimmen 2010	1897	X	CNS	Vestibular Schwannoma	LQ (basic)	None	Local control	X	X	1.77
[29]-1 Maciejewski 1989	175	T1N0M0-T3N3M0	Head & Neck	Squamous cell carcinoma	LQ+RPP	Logistic	Local control	X	X	-9.35
[29]-2 Maciejewski 1989	210	T1N0M0-T3N3M0	Head & Neck	Squamous cell carcinoma	LQ+RPP	Logistic	Local control	X	X	-14.08
[29]-3 Maciejewski 1989	72	T1N0M0-T3N3M0	Head & Neck	Squamous cell carcinoma	LQ+RPP	Logistic	Local control	X	X	7.25
[29]-4 Maciejewski 1989	41	T1N0M0-T3N3M0	Head & Neck	Squamous cell carcinoma	LQ+RPP	Logistic	Local control	X	X	6.62
[9]-1 Barton 1992	327	T2	Head & Neck	Squamous cell carcinoma	LQ+RPP	Logistic	Local control	X	X	-4.6
[9]-2 Barton 1992	1012	T1-T4	Head & Neck	Squamous cell carcinoma	LQ+RPP	Logistic	Local control	X	X	-42.6
[9]-3 Barton 1992	1012	T1-T4	Head & Neck	Squamous cell carcinoma	LQ+RPP	Logistic	Local control	X	X	-59.4
[9]-4 Barton 1992	208	T1-T2	Head & Neck	Squamous cell carcinoma	LQ+RPP	Logistic	Local control	X	X	23
[9]-5 Barton 1992	247	T3-T4	Head & Neck	Squamous cell carcinoma	LQ+RPP	Logistic	Local control	X	X	27.2
[9]-6 Barton 1992	230	T3-T4	Head & Neck	Squamous cell carcinoma	LQ+RPP	Logistic	Local control	X	X	-7.6
[25] Hendry 1992	4668	T1-T4	Head & Neck	Squamous cell carcinoma	LQ+RPP	Poisson	Local control	0.033	-0.0014	-23.6
[40]-1 Rezvani 1993	470	T1	Head & Neck	Squamous cell carcinoma	LQ+RPP	Logistic	Local control	0.014	0.001	14
[40]-2 Rezvani 1993	195	T3	Head & Neck	Squamous cell carcinoma	LQ (basic)	Logistic	Local control	0.016	0.017	0.94
[40]-3 Rezvani 1993	170	T2	Head & Neck	Squamous cell carcinoma	LQ (basic)	Logistic	Local control	0.021	-0.002	-10.5
[40]-4 Rezvani 1993	470	T1	Head & Neck	Squamous cell carcinoma	LQ (basic)	Logistic	Local control	0.023	0.001	23
[40]-5 Rezvani 1993	470	T1	Head & Neck	Squamous cell carcinoma	LQ+RPP	Logistic	Local control	0.025	0.001	25
[40]-6 Rezvani 1993	470	T1	Head & Neck	Squamous cell carcinoma	LQ+RPP	Logistic	Local control	0.026	0.001	26
[40]-7 Rezvani 1993	195	T3	Head & Neck	Squamous cell carcinoma	LQ+RPP	Logistic	Local control	0.106	0.008	13.25
[40]-8 Rezvani 1993	195	T3	Head & Neck	Squamous cell carcinoma	LQ+RPP	Logistic	Local control	0.107	0.008	13.38
[40]-9 Rezvani 1993	170	T2	Head & Neck	Squamous cell carcinoma	LQ+RPP	Logistic	Local control	0.108	0.006	18

[40]-10 Rezvani 1993	195	T3	Head & Neck	Squamous cell carcinoma	LQ+RPP	Logistic	Local control	0.108	0.008	13.5
[40]-11 Rezvani 1993	170	T2	Head & Neck	Squamous cell carcinoma	LQ+RPP	Logistic	Local control	0.11	0.006	18.33
[40]-12 Rezvani 1993	170	T2	Head & Neck	Squamous cell carcinoma	LQ+RPP	Logistic	Local control	0.117	0.004	29.25
[41]-1 Roberts 1993	4668	T1-T4	Head & Neck	Squamous cell carcinoma	LQ+RPP	Poisson	Local control	0.033	-0.0014	-23.6
[41]-2 Roberts 1993	4668	T1-T4	Head & Neck	Squamous cell carcinoma	LQ+RPP	Poisson	Local control	0.034	-0.0015	-22.7
[42]-1 Roberts 1994	461	T1	Head & Neck	Squamous cell carcinoma	LQ+RPP	Poisson	Local control	0.049	0.0056	8.75
[42]-2 Roberts 1994	180	T3	Head & Neck	Squamous cell carcinoma	LQ+RPP	Poisson	Local control	0.056	0.0038	14.74
[42]-3 Roberts 1994	187	T2	Head & Neck	Squamous cell carcinoma	LQ+RPP	Poisson	Local control	0.092	-0.0011	-83.64
[68] Withers 1995	641	T1N0-T4N3	Head & Neck	Squamous cell carcinoma	LQ+RPP	Cox	Local control	X	X	-14.7
[16]-1 Chappel 1995	766	T1N0M0-T3N0M0	Head & Neck	Squamous cell carcinoma	LQ+RPP	Logistic	Local control	0.07	0.0031	22.5
[16]-2 Chappel 1995	145	T1N0M0-T4N0M0	Head & Neck	Squamous cell carcinoma	LQ+RPP	Logistic	Local control	0.0793	0.0085	9.33
[49] Slevin 1992	496	T2-T3	Head & Neck	Unspecified	LQ+RPP	Poisson	Local control	0.22	-0.0149	-14.8
[44]-1 Robertson 1993	24	T3N0M0-T4N0M0	Head & Neck	Unspecified	LQ+RPP	Poisson	Local control	0.023	0.012	1.9
[44]-2 Robertson 1993	48	T2N0M0	Head & Neck	Unspecified	LQ+RPP	Poisson	Local control	0.159	0.011	14.6
[44]-3 Robertson 1993	95	T1N0M0	Head & Neck	Unspecified	LQ+RPP	Poisson	Local control	0.187	0.014	13.8
[50]-1 Stuschke 1995	321	T2N0M0-T3N1M0	Head & Neck	Unspecified	LQ (basic)	None	TCD50	X	X	19.7
[50]-2 Stuschke 1995	321	T2N0M0-T3N1M0	Head & Neck	Unspecified	LQ (basic)	None	TCD50	X	X	9.9
[46] Saarilahti 1998	117	T1	Head & Neck	Unspecified	LQ+RPP	Logistic	Local control	0.182	0.123	1.48
[45] Robertson 1998	395	T1N0-T4N1	Head & Neck	Unspecified	LQ+RPP	Cox	Local control	0.03	0.001	30
[51] Stuschke 1999	1489	T1N0-T4N3	Head & Neck	Unspecified	LQ (basic)	None	TCD50	X	X	10.64
[55] Thames 1986	58	X	Liposarcoma	Liposarcoma	LQ (basic)	None	Local control	X	X	0.4
[54] Tai 2008	321	T3-T4	Liver	Hepatocellular carcinoma & Cholangiocarcinoma	LQ+RPP	Poisson	Survival	0.037	0.0028	13.4
[52] Stuschke 2010	2038	I-III	Lung	Non small cell lung carcinoma	LQ (basic)	Logistic	Local control	X	X	8.2
[47] Santiago 2016	2319	T1-T2	Lung	Non small cell lung carcinoma	LQ (basic)	Logistic	Local control	X	X	3.9

[22] Geh 2006	1284	X	Oesophagus	Adenocarcinoma & Squamous cell carcinoma	LQ+RPP	Logistic	Pathological complete response	0.04	0.008	4.9
[13] Brenner 1999	367	T1N0-T3N1	Prostate	Adenocarcinoma	LQ (basic)	Poisson	bNED	0.036	0.024	1.5
[14]-1 Brenner 2000*	367	T1N0-T3N1	Prostate	Adenocarcinoma	LQ + heterogeneity	Poisson	bNED	X	X	2.1
[27] King 2000	367	T1N0-T3N1	Prostate	Adenocarcinoma	LQ + heterogeneity	Poisson	bNED	0.346	0.0698	4.96
[21] Fowler 2001*	1450	T2	Prostate	Adenocarcinoma	LQ+RP	Poisson	bNED	0.0391	0.0263	1.49
[15] Brenner 2002*	192	T1-T3	Prostate	Adenocarcinoma	LQ (basic)	Poisson	bNED	0.026	0.0217	1.2
[65] Wang 2003	142	T1-T3	Prostate	Adenocarcinoma	LQ+RP+RPP	Poisson	bNED	0.14	0.0452	3.1
[64] Wang 2003*	1552	X	Prostate	Adenocarcinoma	LQ+RP+RPP	Poisson	bNED	0.15	0.0484	3.1
[17] Chappel 2004*	1450	T2	Prostate	Adenocarcinoma	LQ+RP	Poisson	bNED	0.0317	0.0263	1.44
[11]-1 Bentzen 2005	330	X	Prostate	Adenocarcinoma	LQ (basic)	None	bNED	X	X	8.3
[11]-2 Bentzen 2005	936	T1-T2	Prostate	Adenocarcinoma	LQ (basic)	None	bNED	X	X	1.12
[69] Yeoh 2006	217	T1N0M0-T2N0M0	Prostate	Adenocarcinoma	LQ (basic)	None	bNED	X	X	2.3
[18]-1 Dasu 2007	705	T1N0M0-T4N0M0	Prostate	Adenocarcinoma	LQ (basic)	None	bNED	X	X	1.3
[18]-2 Dasu 2007	282	T1N0M0-T4N0M0	Prostate	Adenocarcinoma	LQ (basic)	None	bNED	X	X	2.4
[67]-1 Williams 2007	3571	T1-T4	Prostate	Adenocarcinoma	LQ (basic)	Cox	bNED	X	X	3.7
[67]-2 Williams 2007*	3756	T1-T4	Prostate	Adenocarcinoma	LQ (basic)	Cox	bNED	X	X	2.6
[67]-3 Williams 2007*	3756	T1-T4	Prostate	Adenocarcinoma	LQ (basic)	Cox	bNED	X	X	4.5
[67]-4 Williams 2007*	3756	T1-T4	Prostate	Adenocarcinoma	LQ (basic)	Cox	bNED	X	X	7.1
[31]-1 Nickers 2010*	328	T1-T3	Prostate	Adenocarcinoma	LQ+RP	None	bNED	X	X	3.41
[31]-2 Nickers 2010*	328	T1-T3	Prostate	Adenocarcinoma	LQ+RP	None	bNED	X	X	5.87
[70] Yeoh 2011	96	T1N0M0-T2N0M0	Prostate	Adenocarcinoma	LQ (basic)	None	bNED	X	X	0.65
[61]-1 Valdagni 2011	84	T1-T4	Prostate	Adenocarcinoma	LQ (basic)	None	bNED	X	X	2.2
[61]-2 Valdagni 2011	148	T1-T4	Prostate	Adenocarcinoma	LQ (basic)	None	bNED	X	X	5.4
[61]-3 Valdagni 2011	84	T1-T4	Prostate	Adenocarcinoma	LQ (basic)	None	bNED	X	X	9

[61]-4 Valdagni 2011	84	T1-T4	Prostate	Adenocarcinoma	LQ (basic)	None	bNED	X	X	0.9
[61]-5 Valdagni 2011	148	T1-T4	Prostate	Adenocarcinoma	LQ (basic)	None	bNED	X	X	3.1
[61]-6 Valdagni 2011	84	T1-T4	Prostate	Adenocarcinoma	LQ (basic)	None	bNED	X	X	7.9
[37] Proust-Lima 2011	5093	T1-T4	Prostate	Adenocarcinoma	LQ (basic)	Other	Slope of PSA rise	0.0062	0.004	1.55
[28] Leborgne 2012	274	X	Prostate	Adenocarcinoma	LQ (basic)	None	bNED	X	X	1.86
[19]-1 Dasu 2012	803	X	Prostate	Adenocarcinoma	LQ (basic)	Logistic	bNED	X	X	0.6
[19]-2 Dasu 2012	996	X	Prostate	Adenocarcinoma	LQ (basic)	Logistic	bNED	X	X	1.2
[19]-3 Dasu 2012	1024	X	Prostate	Adenocarcinoma	LQ (basic)	Logistic	bNED	X	X	1.1
[19]-4 Dasu 2012	803	X	Prostate	Adenocarcinoma	LQ+RPP	Logistic	bNED	X	X	0.6
[19]-5 Dasu 2012	996	X	Prostate	Adenocarcinoma	LQ+RPP	Logistic	bNED	X	X	1.1
[19]-6 Dasu 2012	1024	X	Prostate	Adenocarcinoma	LQ+RPP	Logistic	bNED	X	X	1.6
[19]-7 Dasu 2012	1315	X	Prostate	Adenocarcinoma	LQ (basic)	Logistic	bNED	X	X	1
[19]-8 Dasu 2012	2631	X	Prostate	Adenocarcinoma	LQ (basic)	Logistic	bNED	X	X	1.3
[19]-9 Dasu 2012	2561	X	Prostate	Adenocarcinoma	LQ (basic)	Logistic	bNED	X	X	1.7
[19]-10 Dasu 2012	1315	X	Prostate	Adenocarcinoma	LQ+RPP	Logistic	bNED	X	X	1.3
[19]-11 Dasu 2012	2631	X	Prostate	Adenocarcinoma	LQ+RPP	Logistic	bNED	X	X	1.9
[19]-12 Dasu 2012	2561	X	Prostate	Adenocarcinoma	LQ+RPP	Logistic	bNED	X	X	1.2
[30]-1 Miralbell 2012	1405	X	Prostate	Adenocarcinoma	LQ (basic)	Poisson	bNED	0.01	0.0167	0.6
[30]-2 Miralbell 2012	1405	X	Prostate	Adenocarcinoma	LQ (basic)	Poisson	bNED	0.019	0.0136	1.4
[30]-3 Miralbell 2012	2616	X	Prostate	Adenocarcinoma	LQ (basic)	Poisson	bNED	0.032	0.0229	1.4
[30]-4 Miralbell 2012	2616	X	Prostate	Adenocarcinoma	LQ (basic)	Poisson	bNED	0.036	0.0212	1.7
[30]-5 Miralbell 2012	1948	X	Prostate	Adenocarcinoma	LQ (basic)	Poisson	bNED	0.041	0.0293	1.4
[30]-6 Miralbell 2012	1948	X	Prostate	Adenocarcinoma	LQ (basic)	Poisson	bNED	0.044	0.0275	1.6
[30]-7 Miralbell 2012	1405	X	Prostate	Adenocarcinoma	LQ+RPP	Poisson	bNED	0.051	0.0128	4
[30]-8 Miralbell 2012	2616	X	Prostate	Adenocarcinoma	LQ+RPP	Poisson	bNED	0.078	0.0195	4
[30]-9 Miralbell 2012	1948	X	Prostate	Adenocarcinoma	LQ+RPP	Poisson	bNED	0.098	0.0245	4
[63]-1 Vogelius 2013	1965	X	Prostate	Adenocarcinoma	LQ (basic)	None	bNED	X	X	-0.07
[63]-2 Vogelius 2013	1965	X	Prostate	Adenocarcinoma	LQ+RPP	None	bNED	X	X	0.58
[63]-3 Vogelius 2013	1797	X	Prostate	Adenocarcinoma	LQ (basic)	None	bNED	X	X	0.47

[63]-4 Vogelius 2013	1797	X	Prostate	Adenocarcinoma	LQ+RPP	None	bNED	X	X	1.93
[35]-1 Pedicini 2013	X	X	Prostate	Adenocarcinoma	LQ+RPP	Poisson	bNED	0.14	0.044	3.18
[35]-2 Pedicini 2013	X	X	Prostate	Adenocarcinoma	LQ+RPP	Poisson	bNED	0.15	0.048	3.12
[35]-3 Pedicini 2013	X	X	Prostate	Adenocarcinoma	LQ+RPP	Poisson	bNED	0.15	0.058	2.59
[12]-1 Boonstra 2016	289	T1N0M0-T2N0M0	Prostate	Adenocarcinoma	LQ (basic)	Other	PSA	0.024	0.0031	29.9
[12]-2 Boonstra 2016	289	T1N0M0-T2N0M0	Prostate	Adenocarcinoma	LQ (basic)	Other	PSA	0.055	0.0071	6.5
[12]-3 Boonstra 2016	289	T1N0M0-T2N0M0	Prostate	Adenocarcinoma	LQ (basic)	Other	PSA	0.036	0.0047	7.8
[12]-4 Boonstra 2016	289	T1N0M0-T2N0M0	Prostate	Adenocarcinoma	LQ (basic)	Other	PSA	0.014	0.0018	10.7
[12]-5 Boonstra 2016	289	T1N0M0-T2N0M0	Prostate	Adenocarcinoma	LQ (basic)	Other	PSA	X	X	7.7
[12]-6 Boonstra 2016	289	T1N0M0-T2N0M0	Prostate	Adenocarcinoma	LQ+RPP	Other	PSA	X	X	6.8
[12]-7 Boonstra 2016	289	T1N0M0-T2N0M0	Prostate	Adenocarcinoma	LQ (basic)	Cox	bNED	X	X	18
[53]-1 Suwinski 2007	168	T1N0M0-T4N1M0	Rectum	Adenocarcinoma	LQ (basic)	Cox	Locoregional control	0.265	0.054	4.9
[53]-2 Suwinski 2007	168	T1N0M0-T4N1M0	Rectum	Adenocarcinoma	LQ+RPP	Poisson	Locoregional control	0.335	0.03	11.1
[53]-3 Suwinski 2007	168	T1N0M0-T4N1M0	Rectum	Adenocarcinoma	LQ (basic)	Poisson	Locoregional control	0.339	0.067	2.66
[59] Timmerman 2002	490	X	Rhabdo-myosarcoma	Rhabdomyosarcoma	LQ (basic)	None	Relapse free survival / disease free survival	X	X	2.8
[60]-1 Trott 1984	909	X	Skin	Basal-cell carcinoma & Squamous cell carcinoma	LQ (basic)	None	TCD90	X	X	13.8
[60]-2 Trott 1984	909	X	Skin	Basal-cell carcinoma & Squamous cell carcinoma	LQ (basic)	None	TCD50	X	X	9.5
[60]-3 Trott 1984	909	X	Skin	Basal-cell carcinoma & Squamous cell carcinoma	LQ (basic)	None	TCD37	X	X	8.8
[56] Thames 1990	784	X	Skin	Basal-cell carcinoma & Squamous cell carcinoma	LQ (basic)	None	Local control	X	X	8.5

[14]-2 Brenner 2000	784	X	Skin	Basal-cell carcinoma & Squamous cell carcinoma	LQ + heterogeneity	Poisson	Local control	X	X	9.5
[5] Dale 1985	X	X	Skin	Carcinoma (NOS)	LQ+RP	None	Unspecified	X	X	7.6
[32] Overgaard 1986	114	X	Skin	Melanoma	LQ (basic)	None	TCD50	X	X	2.5
[33] Overgaard 1986	618	X	Skin	Melanoma	LQ (basic)	None	TCD50	X	X	2.5
[10] Bentzen 1989	239	X	Skin	Melanoma	LQ (basic)	Poisson	Local control	0.0053	0.0092	0.57
[57] Thames 1995	126	X	Skin	Melanoma	LQ (basic)	None	Complete response	X	X	7.2

*Included data of patients treated with brachytherapy as part of the treatment.

Nomenclature

Histologies
NOS: not otherwise specified

LQ model
RP: repair
RPP: repopulation

N.B. Occasionally the same study and patient group appears multiple times in the table with, other than different outcomes, no apparent difference. This occurs when authors repeatedly analyzed the same data using slightly different analysis methods, but those differences were not reflected in the variables selected for extraction. E.g. Barton (1992) performed two similar analyses on his cohort of 1012 patients, the only difference was that in the second analysis ([9]-3) additional factors were included to correct for stage and (sub)site.

Additional file 1: Table S5 – Common LQ models and TCP models

LQ models

Table S5.1
Types of LQ models and their formulae.

LQ models	Formula
Basic	$SF = Exp[-\alpha \cdot D - \beta \cdot d \cdot D]$
Basic + repopulation	$SF = Exp[-\alpha \cdot D - \beta \cdot d \cdot D + Ln[2]\frac{T - T_k}{T_{pot}}]$
Basic + incomplete repair	$SF = Exp[-\alpha \cdot D_T - \beta \cdot d \cdot D - \frac{2 \cdot \beta \cdot R^2}{\mu}\left\{T_{Pr} - \frac{1 - \exp[-\mu \cdot T_{pr}]}{\mu}\right\}]$
Basic + incomplete repair + repopulation	$SF = Exp\left[-\alpha \cdot D_T - \beta \cdot d \cdot D - \frac{2 \cdot \beta \cdot R^2}{\mu}\left\{T_{pr} - \frac{1 - \exp[-\mu \cdot T_{pr}]}{\mu}\right\} + Ln[2]\frac{T - T_k}{T_{pot}}\right]$
Basic + heterogeneity	$SF_i = \exp[-\alpha_i \cdot D - \beta_i \cdot d \cdot D]$

Nomenclature
SF: the fraction of surviving cells
α, β: radiosensitivity parameters
D: total unprotracted dose
D_T: total dose including protracted dose
d: the fraction dose
T: the overall treatment time
T_k: the delay of accelerated repopulation
T_{pot}: the potential doubling time
T_{Pr}: the total time over which the protracted dose is delivered
R: the dose rate for the protracted irradiation
μ: the repair constant for potentially lethal damage repair.

The formulae provided here (Table S5.1) are examples, though several ways of expressing the same terms exist. Expressions in the included papers are therefore not necessarily the same as provided here.

In the LQ model incorporating (intratumor) heterogeneity, the radiosensitivity parameters α_i and β_i represents the radiosensitivity in subvolume i of the tumor, and SF_i represents the survival fraction in that subvolume. The parameters α_i and β_i are commonly assumed to follow a normal or log-normal distribution.

TCP models

If two different fractionation schemes prove to be equivalent, these can be used to estimate α/β. However, to estimate separate α and β values, the surviving fraction predicted by the LQ model needs to be linked to a clinical outcome using explicit models, which are shown in Table S5.2.

Table S5.2
TCP models and their formulae.

TCP models	Formula
No explicit model	None (uses two equivalent treatment schemes to derive α/β)
Poisson TCP	$TCP = Exp[-N_0 \cdot SF]$
Logistic TCP	$TCP = \frac{1}{1 + N_0 \cdot SF}$
Cox proportional hazards	$\lambda(t) = \lambda_0(t) \cdot N_0 \cdot SF$

Other	Some custom formula was used to relate the LQ model to a clinical outcome.

The expression used for the surviving fraction depends on which type of LQ model was used.

Instead of N_0 (which for the Poisson model is generally interpreted as the number of clonogenic cells at the start of treatment), a parametrization $k = Ln[N_0]$ is often used so that the constant k can be included in the linear-quadratic part of the survival formula. E.g. for a combination of a Poisson TCP model and basic LQ model, the TCP model is often written as $TCP = Exp[-Exp[k - \alpha \cdot D - \beta \cdot d \cdot D]]$ rather than $TCP = Exp[-N_0 \cdot Exp[-\alpha \cdot D - \beta \cdot d \cdot D]]$. Mathematically, these expressions are identical.

In the Cox proportional hazards model, $\lambda(t)$ represents the hazard function and $\lambda_0(t)$ is the baseline hazard function.

Additional file 1: Table S6 – Heterogeneity with/without studies including brachytherapy

Table S6.1
Heterogeneity in α/β, α and β estimates when grouped by site. I^2 values excluding those studies that included data from patients treated with brachytherapy are also shown (I^2, no BT). A dash indicates that there were insufficient studies (i.e. less than two) for calculating I^2 for that combination of outcome (α/β, α and β) and tumor site.

	α/β		α		β	
**Tumour site**	**I^2 (%)**	**I^2, no BT (%)**	**I^2 (%)**	**I^2, no BT (%)**	**I^2 (%)**	**I^2, no BT (%)**
Bladder	0	-	0	-	0	-
Breast	0	0	66	66	-	-
CNS	60	60	0	-	-	-
Head & Neck	87	87	95	95	84	84
Lung	88	88	-	-	-	-
Prostate	94	92	99	99	99	99
Rectum	0	0	0	0	0	0
Skin	97	97	-	-	-	-

Table S6.2
Heterogeneity in α/β, α and β estimates when grouped by histology. I^2 values excluding those studies that included data from patients treated with brachytherapy are also shown (I^2, no BT). A dash indicates that there were insufficient studies (i.e. less than two) for calculating I^2 for that combination of outcome (α/β, α and β) and tumor histology.

	α/β		α		β	
**Tumour histology**	**I^2 (%)**	**I^2, no BT (%)**	**I^2 (%)**	**I^2, no BT (%)**	**I^2 (%)**	**I^2, no BT (%)**
Adenocarcinoma	94	91	99	99	98	99
Glioma	0	0	0	0	-	-
Melanoma	0	0	-	-	-	-
Meningioma	0	0	-	-	-	-
Non small cell lung carcinoma	88	88	-	-	-	-
Squamous cell carcinoma	90	90	95	95	87	87
Transitional cell carcinoma	0	-	0	-	0	-
Unspecified (breast)	0	0	71	71	-	-
Unspecified (head & neck)	0	0	65	65	40	40
Vestibular Schwannoma	0	0	-	-	-	-
